# Targeted sequencing of high-density SNPs provides an enhanced tool for forensic applications and genetic landscape exploration in Chinese Korean ethnic group

**DOI:** 10.1186/s40246-023-00541-0

**Published:** 2023-11-27

**Authors:** Qiong Lan, Yifeng Lin, Xi Wang, Xi Yuan, Chunmei Shen, Bofeng Zhu

**Affiliations:** 1 Microbiome Medicine Center, Department of Laboratory Medicine, Zhujiang Hospital, Southern Medical University, Guangzhou, China; 2 Guangzhou Key Laboratory of Forensic Multi-Omics for Precision Identification, School of Forensic Medicine, Southern Medical University, Guangzhou, China; 3 Department of Laboratory Medicine, Nanfang Hospital, Southern Medical University, Guangzhou, China; 4 Key Laboratory of Shaanxi Province for Craniofacial Precision Medicine Research, College of Stomatology, Xi’an Jiaotong University, Xi’an, China

**Keywords:** Next-generation sequencing, Single nucleotide polymorphism, Forensic application, Genetic structure, Chinese Korean ethnic group

## Abstract

**Background:**

In this study, we present a NGS-based panel designed for sequencing 1993 SNP loci for forensic DNA investigation. This panel addresses unique challenges encountered in forensic practice and allows for a comprehensive population genetic study of the Chinese Korean ethnic group. To achieve this, we combine our results with datasets from the 1000 Genomes Project and the Human Genome Diversity Panel.

**Results:**

We demonstrate that this panel is a reliable tool for individual identification and parentage testing, even when dealing with degraded DNA samples featuring exceedingly low SNP detection rates. The performance of this panel for complex kinship determinations, such as half-sibling and grandparent-grandchild scenarios, is also validated by various kinship simulations. Population genetic studies indicate that this panel can uncover population substructures on both global and regional scales. Notably, the Han population can be distinguished from the ethnic minorities in the northern and southern regions of East Asia, suggesting its potential for regional ancestry inference. Furthermore, we highlight that the Chinese Korean ethnic group, along with various Han populations from different regional areas and certain northern ethnic minorities (Daur, Tujia, Japanese, Mongolian, Xibo), exhibit a higher degree of genetic affinities when examined from a genomic perspective.

**Conclusion:**

This study provides convincing evidence that the NGS-based panel can serve as a reliable tool for various forensic applications. Moreover, it has helped to enhance our knowledge about the genetic landscape of the Chinese Korean ethnic group.

**Supplementary Information:**

The online version contains supplementary material available at 10.1186/s40246-023-00541-0.

## Background

In recent decades, short tandem repeats (STRs) have emerged as highly efficient and well-established DNA markers in forensic analyses because of their high genetic polymorphisms, particularly for individual identification and parentage testing cases [1, 2]. The prevailing method for STR genotyping is polymerase chain reaction (PCR)-based capillary electrophoresis (CE) profiling due to its great convenience and cost-effectiveness [3, 4]. However, challenges persist in CE-based STR profiling when dealing with highly degraded, mixed and low-templated DNA in complex cases [5, 6]. These challenges encompass several aspects: (1) it is difficult to determine the minor contributor’s DNA genotype in mixed samples using CE, especially when the ratio of the minor to major donor DNA is lower than 1:10 [7]; (2) the STR loci exhibit poor performance when applied to highly degraded DNA (< 50 bp) because they rely on amplifying large target regions; (3) when STR typing is performed on low-template DNA, there is a heightened risk of unbalanced amplification of STR alleles, as well as the occurrence of random allele drop-out or allele drop-in. Therefore, to further enhance the efficacy of DNA analyses in forensic investigations, there are still many challenges that need to be addressed by forensic scientists. Fortunately, the advent of sequencing technologies has greatly broadened the scope and depth of applications in forensic DNA investigation, transitioning from low-throughput of a limited number of genetic markers to high-throughput sequencing of tens of thousands of genetic markers. This expanded capacity presents novel avenues for resolving intricate forensic scenarios [8, 9]. Single nucleotide polymorphisms (SNPs) have been considered as promising markers for forensic DNA analysis. The major advantages of SNPs over STRs rely on their high prevalence in the human genome, low mutation rate, and small sizes of amplified SNP alleles [10]. Next-generation sequencing (NGS) technology has enabled the concurrent analysis of a large number of SNPs from multiple samples in a single experiment, effectively compensating for the limitations inherent in STR-based method. In previous studies, NGS-based panels have been constructed with SNP markers for various forensic applications [9, 11, 12], thereby providing more possibilities for forensic DNA analysis.

In addition to forensics, advances in NGS technology have also facilitated major initiatives to genotype diverse global populations, including the Human Genome Diversity Panel (HGDP) [13], 1000 Genomes Project (1 KG) [14], Estonian Biocentre Human Genome Diversity Panel [15], and Simons Genome Diversity Project [16]. These projects have established a foundation for documenting the structure and historical backgrounds of various populations on a global scale. East Asia is rich in ethnic, linguistic, and genetic diversity, attracting sustained scholarly attention. The Korean people predominantly reside on the Korean Peninsula. However, it is estimated that about 7.3 million Korean individuals inhabit regions beyond the Korean peninsula, encompassing residences such as China, Japan, the United States, and various other places of the world. In East Asia, most of the research on Korean-speaking populations has predominantly concentrated on the southern region of the Korean Peninsula, and the Chinese Korean ethnic group has remained relatively underrepresented in academic inquiry, particularly in genomic investigations. Therefore, a limited amount of genetic data specific to the Korean population has been collected as part of broader surveys targeting East Asian diversity. Importantly, the Chinese Korean ethnic group is officially recognized as one of the 55 ethnic minorities by the Chinese government, and their population reached 1,702,479 according to the China Statistical Yearbook 2021. The majority of the Chinese Koreans reside in Yanbian Autonomous Korean Prefecture, in close proximity to the Korean peninsula. The geographical proximity between China and the Korean peninsula has engendered migratory flows and cultural exchanges between different ethnic groups in these two regions, inevitably influencing on their genetic backgrounds. Present-day Koreans in South Korea exhibit close genetic relationships to their East Asian counterparts from all perspectives including patrilineal, matrilineal, and biparental, as previously reported [17]. Notwithstanding, the Chinese Korean ethnic group has been relatively overlooked in population genetics investigations. A prevailing limitation lies in the employment of a restricted set of DNA genetic markers in prior studies, casting doubt on the capacity of these markers to provide an exhaustive and profound elucidation of the genetic landscape characterizing the Chinese Korean ethnic group. Therefore, the key question about the genetic background of the Chinese Korean ethnic group remains largely uncharacterized.

Herein, we presented a new panel designed to enable the concurrent amplification of 1993 SNP loci in a single assay for various forensic applications. This new panel is built on the MGISEQ-2000RS platform using targeted sequencing technology, which employs DNA nanospheres and rolling cycle amplification for sequencing, thereby minimizing genotypic errors typically caused by PCR [18, 19]. Participants from the Chinese Korean ethnic group were genotyped using this new panel for a thorough evaluation of its effectiveness for various forensic and population genetic applications. Specifically, the abilities of this panel for individual identification, parentage testing and complex kinship analysis were systematically evaluated with reliable estimations. Furthermore, we merged the data newly generated from our study with previously published datasets (1 KG and HGDP) to obtain a total of 3590 individuals representing major intercontinental populations across eight continents, and performed population genetic analyses on these groups. These efforts shed light on the genetic landscape of the Chinese Koreans from a genomic perspective. This study will help to further enrich the genetic diversity within the Chinese Korean ethnic group and provide new insights into its genetic heritage.

## Methods

### Ethics statement

The Ethics Committee of Southern Medical University and Xi'an Jiaotong University reviewed and approved this study (No. 2019-1039). Additionally, it adhered to the guidelines presented in the revised Declaration of Helsinki from 2000. Before the sample collection, all participants gave their written informed consent.

### Sample collection

A total of 161 unrelated individuals were recruited from the Yanbian Korean Autonomous Region, China, to participate in this study. The participants were identified as Chinese Koreans by referring to the following criteria: (1) Residency in this region for at least three generations and their parents being Chinese Koreans; (2) Absence of intermarriage events with individuals from other ethnic groups (including the Han population) in their family histories; (3) Proficiency in the Korean language as their native tongue. To ensure that all participants were biologically unrelated to each other, we performed data quality control on the genotyping results of all samples by PLINK (version 1.9) software [20]. Subjects found to be biologically related were excluded. Peripheral blood samples of these participants were collected after they were made aware of the purpose of this study and signed the written informed consent. To better preserve the samples, we prepared bloodstains by applying a portion of peripheral blood (about 200 μL) to FTA cards.

### DNA extraction, library preparation, and variants calling

DNA of the 161 Chinese Koreans was extracted from bloodstains on FTA cards using the Magbead Blood Spots DNA Kit from CWBIO in Beijing, China, following the manufacturer's instructions. The MGIEasy Pa-SNPs panel (MGI, Shenzhen, China) was used to prepare the libraries. We added 10 ng of DNA to a centrifuge tube and mixed it with Elution Buffer to make a final volume of 20.5 μL for the experiment. The library preparation process involved two rounds of PCR, which has been detailed explained in a previous study [21]. Following the two rounds of PCR, the products of the second round PCR were cleaned up using DNA Clean Beads, following the manufacturer's instructions. We used the Qubit® dsDNA HS Assay Kit on a Qubit® 4.0 Fluorometer (Thermo Fisher Scientific, Waltham, USA) to determine the quality of purified PCR products. The second-step PCR products needed to have a concentration of at least 5 ng/μL, and the purified products were required to have a final size distribution of 140 to 180 bp for subsequent DNA sequencing.

DNA nanoballs were prepared according to the manufacturer’s instructions with the final concentration being ≥ 8 ng/μL. Then, DNB sequencing was conducted on the MGISEQ-2000RS platform, configured with FCL SE50 + 10 settings. We used the SoaPnuke2 software to analyze the raw data. All reads were aligned to hs37d5 reference sequences using Burrows-Wheeler Aligner software. SNP genotypes were called using the Freebayes software (http://clavius.bc.edu/~erik/freebayes/).

### Forensic application estimations

We used the STRAF (version 2.1) software [22] to calculate various descriptive statistical parameters, including probability of match (PM), power of discrimination (PD), probability of exclusion (PE), observed heterozygosity (H_obs_) and genetic diversity (GD). Besides, the Hardy–Weinberg equilibrium (HWE) test for each SNP locus were also estimated using both PLINK and STRAF, adjusting the *p* (*p* > 0.000025) values for HWE tests following Bonferroni’s correction. The linkage disequilibrium (LD) tests among pairwise SNP loci were assessed using the Haploview (version 2.1) software [23], with LD determined at a *r*^2^ > 0.2. These parameters were used to evaluate the genetic diversities of the 1993 SNPs in the Chinese Korean ethnic group. Additionally, we computed the cumulative probability of discrimination (CPD) values and the cumulative probability of exclusion (CPE) values to confirm the effectiveness of this new panel for individual identification and parentage testing. We used the Familias (version 3.01) software [24] to simulate pairwise relationship tests in parent–child (PC), full-sibling (FS), half-sibling (HS) and grandparent-grandchild (GG) cases in the Chinese Korean ethnic group. The likelihood ratios (LRs) for each kinship hypothesis (H_1_) compared to the values for the unrelated hypothesis (H_2_) were calculated and compared. We used the allele frequencies of the SNPs in the Chinese Korean ethnic group to simulate genotype data for PC, FS, HS and GG relationships and further generated the LR distributions of these kinship cases. For these SNP loci, the mutation rate of 1.29 × 10^–8^ was determined based on a previous study [25].

### Dataset compilation

In addition to the data generated in this study, we assembled previously published public datasets, including the 1 KG and the HGDP datasets, to construct a comprehensive dataset for population genetic analyses. The composite dataset contained genotype data of present-day human populations representing eight continents, namely Africa, Europe, East Asia, Central South Asia, South Asia, America, Middle East, and Oceania. To ensure the robustness of our estimates, the method requires an SNP set with a minor allele frequency higher than 5%, and further mandated that the SNPs under consideration exhibit independence from each other. Therefore, we pruned the SNP loci using the PLINK software, which resulted in 1706 SNP loci available for subsequent population genetic analyses. The combined dataset consisted of genotype data from 1706 SNPs spanning 76 populations located across eight major global regions. For convenience, the Chinese Korean ethnic group was abbreviated as Korean_C in the data analyses. Detailed information on the geographic origins, names and abbreviations of the 75 reference groups can be accessed in Additional file 1: Table S1.

### Principal components analysis

Population-level principal component analysis (PCA) was performed utilizing the smartpca program in the EIGENSOFT package [26] for the Chinese Korean ethnic group and 75 present-day reference populations from the 1 KG and HGDP. We adopted default settings except for lsqproject: YES, numoutlieriter: 0, and shrinkmode: YES.

To perform the individual-level PCA, we utilized the GraphPad Prism (version 9.4.1) software to compute the PCs that explained the majority of variances within the compiled dataset. Notably, the individual-level PCA was performed stepwise to distinguish between different population groups. Initially, all 76 populations from eight intercontinental geographic regions were employed to construct the principal component space on which we projected all individuals. Subsequently, we retained populations from Asia (i.e., East Asia, South Asia, and Central South Asia), along with those from the Middle East, to generate a more distinct distribution of these geographically closed populations. Lastly, we divided these populations, all of East Asian ancestry, into Han, Northern minority, and Southern minority groups for PCA to show their distribution patterns. The ggplot2 package in *Rstudio* software was used to visualize PCA results using the first three PCs.

### Population structure analysis

We used the ADMIXTURE (version 1.3.0) software [27] to analyze the genetic structures of 76 populations, comprising a total of 3590 individuals, and estimate individual ancestries through maximum likelihood estimation. To determine the best hypothetical ancestral population values (*K*) with the lowest cross-validation (CV) errors, we ran ADMIXTURE with *K* values ranging from 2 to 10. We conducted 20 algorithm iterations for each *K* value. The population structures were visualized using the pophelper packages in *Rstudio* and plotted the replicate of each *K*.

### ***F***_ST_ and ***f*** statistics

In the combined dataset, the *F*_ST_ matrix among different populations was evaluated using the smartpca function in EIGENSOFT (version 7.2.1) software [26]. The "fstonly: YES" option was used for this assessment.

The f-statistic framework was used to explore the genetic relationships between the Chinese Korean ethnic group and the reference populations by computing the outgroup-*f*_3_ and *f*_4_ statistic tests using the “q3pop” and “qpDstat” commands in the AdmixTools2 packages [28] in the *R* program, respectively. The outgroup-*f*_3_ statistic was computed as *f*_3_ (Mbuti; Korean_C, X) to explore the shared genetic drifts between the Chinese Korean ethnic group and the reference populations, where X represented various populations from the combined dataset (except for the Korean_C and Mbuti populations). Higher *f*_3_ values indicated that more genetic drifts had occurred between the Korean_C group and X, suggesting their higher genetic similarities. The pairwise outgroup-*f*_3_ results were then presented with a heatmap using the *R* package ggplot2.

The *f*_4_ statistics were measured using the ordered set (Mbuti, Korean_C; X, Y), where X represented the East Asia Han populations (Northern Han, Beijing Han, and Southern Han) and Y represented the non-Han populations (Cambodian, Dai, Daur, Hezhen, Japanese, Lahu, Miao, Mongolian, Naxi, Oroqen, She, Tu, Tujia, Vietnam, Xibo, Yakut, Yi) in East Asia. In this test, a significant positive *f*_4_ statistic indicated gene flow between Korean_C and Y, while significant negative *f*_4_ statistics suggested potential gene flow between Korean_C and X.

### Maximum-likelihood tree

The phylogenetic relationships within these populations were inferred based on different algorithms. First, a population-level unrooted neighbor-joining (NJ) tree was constructed with the *F*_ST_ values between pairwise populations using the MEGA software (version 11.0) [29]. Next, the phylogenetic tree of the overall 3590 individuals was built on their genotype data with the Tassel software (version 5.2.89) [30]. These two trees were then visualized using the online Chiplot (https://www.chiplot.online/) and the iTOL (https://itol.embl.de/) tools, respectively. Besides, we also used the TreeMix (version 2.1) software [31] to reconstruct the phylogeny of these populations coupled with the potential admixture events. In the TreeMix analysis, the Mbuti population was used as an outgroup to root the tree. The gene flow among these populations were tested by assuming one to eight migration edges and running five algorithm iterations for each. The scripts for data analysis with TreeMix were available on GitHub (https://github.com/carolindahms/TreeMix) and were kindly provided by the developers. We used the 'OptM' package in *R* to estimate the optimal number of admixture events.

## Results

### Data quality control

We manually checked the sequencing data quality to ensure a reliable estimation of the forensic efficiencies for the analyzed SNP loci in the Chinese Korean ethnic group. This process involved a thorough evaluation of the newly generated dataset, including both per-sample and per-marker examinations. We removed samples with mapping rates < 100% and excluded SNP loci with allele calling rates < 100% to generate a comprehensive dataset for subsequent analyses. Eventually, four samples and 47 SNPs were excluded, leaving a total of 157 samples and 1946 SNP loci (accounted for 97.6% of the total SNP loci) for further forensic analyses. These 1946 SNP loci spanned across the 22 autosomes in the human genome (Additional file 1: Fig. S1A).

To determine whether there were any biologically related participants in this study, we performed the kinship analysis among the 157 Korean individuals. We showed that none of the 157 individuals shared close relatedness with each other. Consequently, 157 unrelated Korean individuals were recruited for subsequent analyses of forensic and population genetic analyses. The sequencing depth of these SNPs varied from 106.85 ± 39.62×  to 20,376.01 ± 6654.35×, with most of the sequencing depth concentrated between 1000×  and 5000× (Additional file 1: Fig. S1B). We also presented the SNPs with sequencing depths below 500 × in a boxplot, as shown in Additional file 1: Fig. S1C. The results showed that the minimal sequencing depth for the all 1946 SNP loci exceeded 50×. Furthermore, the histogram (Additional file 1: Fig. S1D) showed that the majority of heterozygous SNP loci had an average coverage ratio (ACR) ranging from 0.6538 to 0.9538. Overall, the results suggested that high-quality SNP data have been generated in the Chinese Korean ethnic group with the new NGS-based panel, further ensuring the reliability of subsequent analyses.

### Forensic performance of the 1946 SNP loci in the Chinese Korean ethnic group

For the HWE tests of the 1946 SNPs, 13 diallelic SNPs disconformed to HWE after Bonferroni correction (*p* < 0.000025). The results of LD tests for pairwise SNP loci showed that only 14 pairs (14/1048575 = 0.0013%) of SNP loci deviated from LD in the Chinese Korean ethnic group.

The statistical parameters for the 1946 SNPs were computed as a crucial step in providing informative recommendations for evaluating the panel's potential for forensic use in the Chinese Korean ethnic group. The 1946 SNP loci consisted of 1906 diallelic SNPs and 40 tri-allelic SNPs. We excluded 13 diallelic SNP loci for being completely homozygosity and heterozygosity in the Chinese Korean ethnic group before visualizing the statistical parameters of these SNP loci. Overall, these SNP loci displayed high polymorphisms in the Chinese Korean ethnic group (Additional file 1: Fig. S2A). In the diallelic SNP set, PD values ranged from 0.0252 to 0.5016; GD values varied from 0.0126 to 0.5016; PM values were in the range of 0.3412 to 0.9748; H_obs_ values varied from 0.0064 to 0.9873; and PE values spanned from 0.0002 to 0.9744. In the tri-allelic SNP set, PD values of these SNPs ranged from 0.3042 to 0.8034; GD values varied from 0.1944 to 0.6602; PM values spanned from 0.1966 to 0.6958; H_obs_ values were in the range of 0.1465 to 0.9172; PE values ranged from 0.0169 to 08307. Overall, the medians of PD, GD, PM, H_obs_, and PD values were 0.5529, 0.4154, 0.4471, 0.4164, 0.1368 and 0.6916, 0.5410, 0.3084, 0.5518, 0.2601 in the diallelic; and tri-allelic SNP sets, respectively, indicating that the tri-allelic SNP loci were genetically more polymorphic than diallelic SNP loci.

Additionally, we estimated the distribution patterns of 1-CPD and 1-CPE values while incrementally incorporating SNP loci in descending order. Generally, both 1-CPD and 1-CPE values gradually decreased as the number of SNP loci increased. The lowest 1-CPD and 1-CPE values for the 1946 SNPs in the Chinese Korean ethnic group were 3.76E-308 and 2.18E-130, respectively. These results indicated that this panel can be effectively used for individual identification and parentage testing (Additional file 1: Fig. S2B). Moreover, even when the successful detection rate of the SNP loci decreased to 10% (200/1993), the 1-CPD and 1-CPE values could still meet the statistically required identification thresholds for individual identification and parentage testing. For more information on the forensic parameters of these SNPs, referring to Additional file 1: Table S2.

**Fig. 1:**
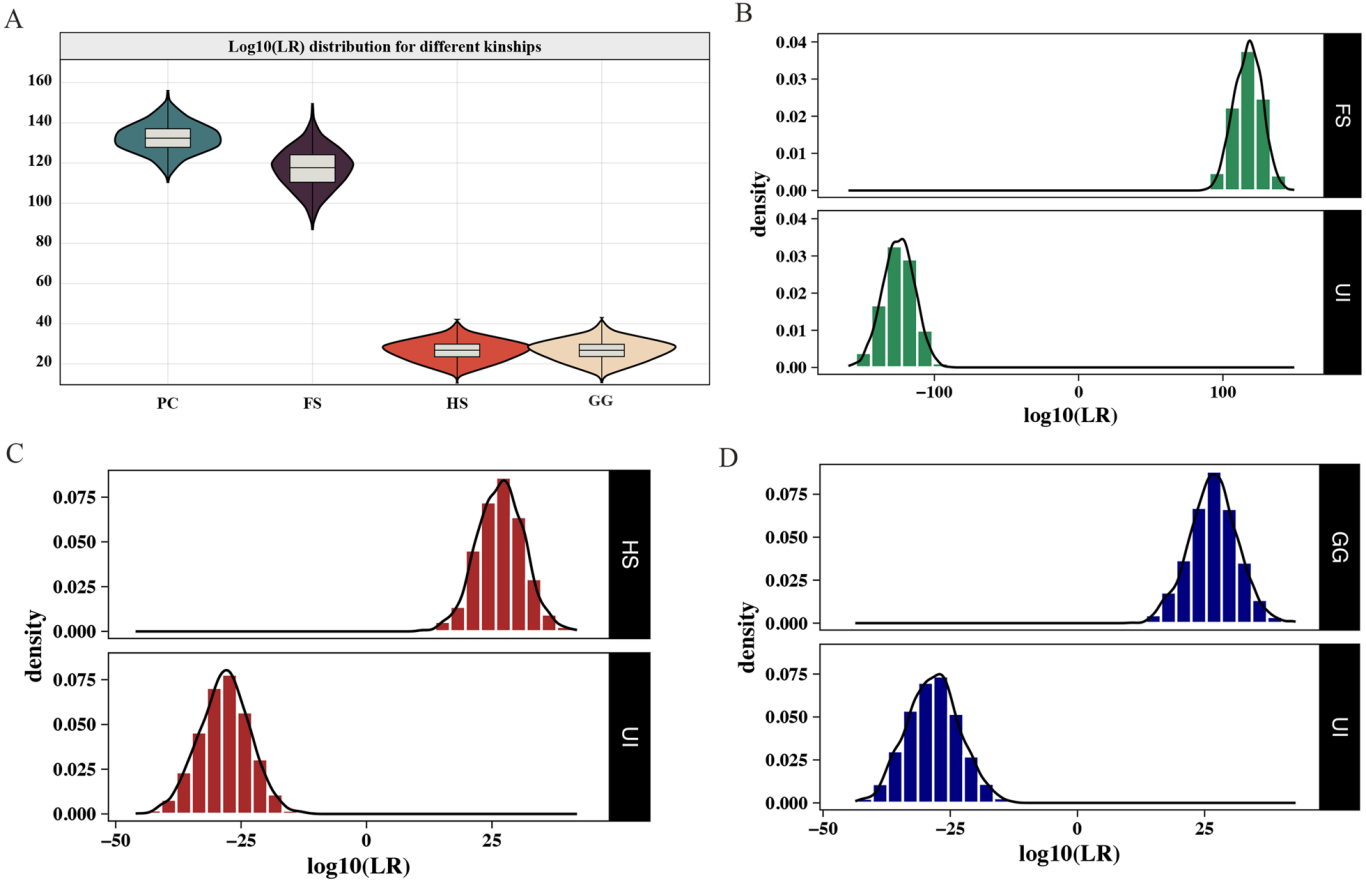
Estimating the efficiencies of the 1946 SNP loci for kinship analyses by simulating different kinships. **A** Box plots displaying the distributions of Log_10_ (LR) for different kinships based on 1946 SNP loci. PC, parent–child pair; FS, full sibling pair; HS, half sibling pair; GG, grandparent-grandchild pair; **B** Log_10_ (LR) distributions for FS and unrelated individuals (UI); **C** Log_10_ (LR) distributions for HS and unrelated individuals (UI); **D** Log_10_ (LR) distributions for GG and unrelated individuals (UI)

Furthermore, we conducted simulations for PC, FS, HS, and GG relationships based on the allele frequencies of the 1946 SNPs in the Chinese Korean ethnic group. Each simulation was repeated 2000 times, and we analyzed the LR for each type of kinship to determine their distribution patterns. As shown in Fig. 1A, significant differences were observed in the log_10_(LR) distribution for first-degree kinship (including PC and FS) and second-degree kinships (encompassing HS and GG). For the first-degree kinships, the log_10_(LR) values for PC and FS were in the range of 110.46 to 155.90 (mean of 132.48 ± 6.90 standard deviation) and 87.02 to 149.38 (mean of 117.31 ± 9.62 standard deviation), respectively. For the second-degree kinships, the Log_10_(LR) values for HS and GG kinships were distributed from 10.65 to 41.95 (mean of 26.71 ± 4.66 standard deviation), and 10.81 to 42.88 (mean of 26.71 ± 4.74 standard deviation), respectively. The distributions of Log_10_(LR) values for FS, HS and GG kinships were entirely distinct from those of unrelated individuals in the simulated kinships (Fig. 1B–D), demonstrating that the new NGS-based panel was potentially useful for identifying first-degree and second-degree kinships in the Chinese Korean ethnic group.

### Informativeness for assignment (***I***_n_) statistics estimated for the 1706 SNPs

**Fig. 2:**
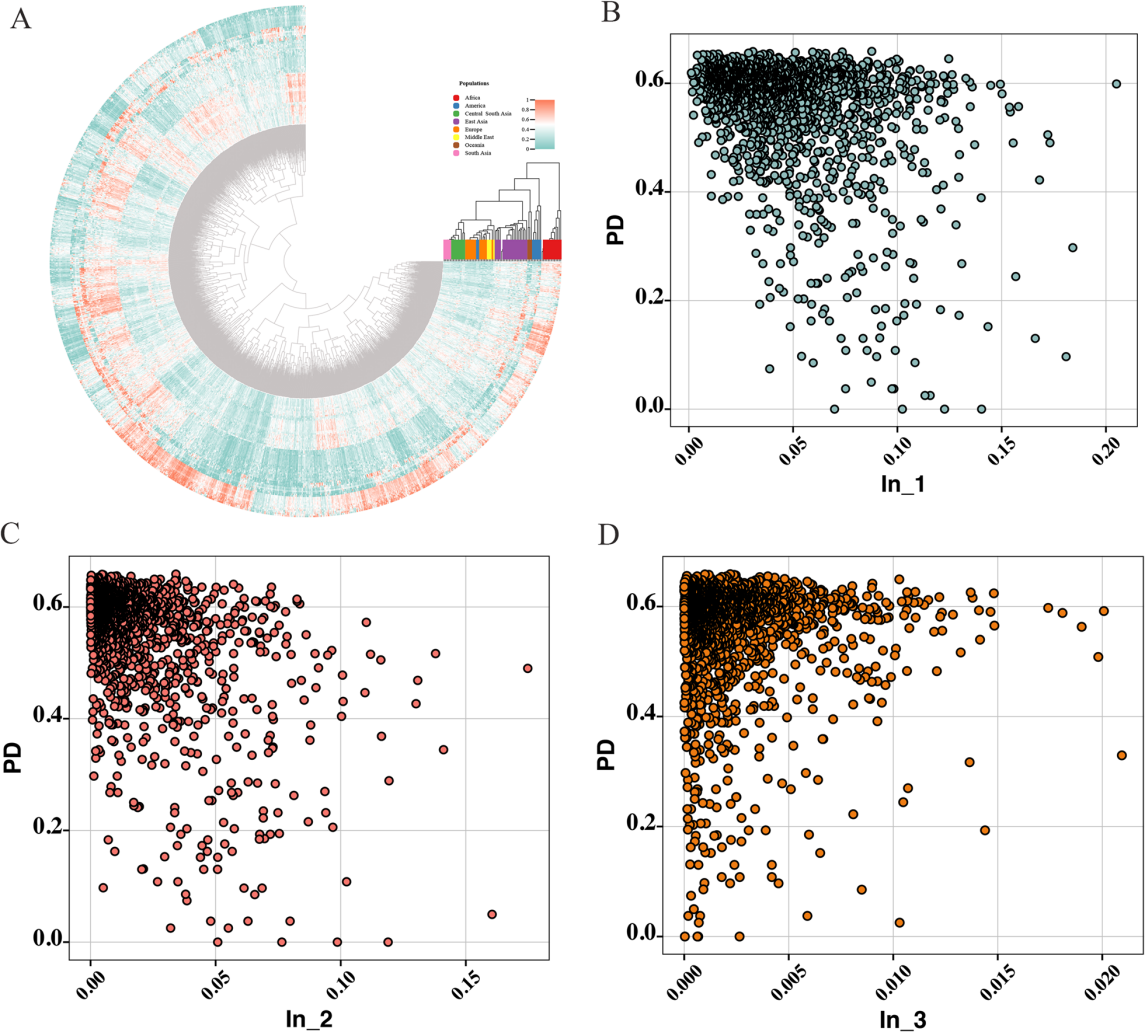
Forensic applications of the 1706 SNP loci in individual identification and ancestry inference. **A** Heatmap of the allele frequencies for the 1706 SNP loci in the 76 populations; **B** Distribution of PD and *I*_n_1_ values of the 1706 SNPs to distinguish among the overall eight intercontinental populations **C** Distribution of PD and *I*_n_2_ values of the 1706 SNPs to distinguish between Asia populations; **D** Distribution of PD and *I*_n_3_ values of the 1706 SNPs to distinguish between East Asia populations. The *I*_n_1_ denotes that the SNP loci are used to distinguish between the overall 76 intercontinental populations. The *I*_n_2_ is calculated to show the efficiency of the SNP locus to distinguish between East Asia, South Asia, Central South Asia and Middle East populations. The *I*_n_3_ is estimated to show the efficiency of the SNP locus in distinguishing between East Asia populations

By incorporating the 75 reference populations into this study, we were able to conduct a comprehensive evaluation of the ancestry inference power for this panel. Initially, we visualized the allele frequencies of the 1706 overlapping SNPs across all 76 populations through a heatmap (Fig. 2A). These results revealed notable differentiation in the allele frequencies of specific SNP loci across various populations, suggesting that these SNP loci could be potentially useful for ancestry inference. Subsequently, we further used the infocalc program to compute the informativeness for assignment (I_n_) statistics to measure the ancestry information content of these SNP loci in distinguishing among different populations. The I_n_ values were calculated to distinguish populations at different levels. I_n_1_ denoted the capacity of the SNP loci to differentiate among the overall 76 intercontinental populations. I_n_2_ quantified the effectiveness of the SNP loci in distinguishing populations from East Asia, South Asia, Central South Asia and Middle East. I_n_3_ specifically gauged their efficiencies in distinguishing East Asian populations. We also generated the scatter plots to provide an intuitive distribution of the forensic efficiencies for these SNP loci, with the X and Y axes demonstrating the I_n_ and PD values of each SNP locus. As shown in Fig. 2B–D, while most SNP loci exhibited superior effectiveness in individual identification, some also displayed potential for ancestry inference. In addition, the number of SNP loci with higher ancestral inference efficiency increased with the geographic separation of the distinguished groups. To support future ancestry inference studies, we also provided the SNP loci with robust ancestry inference efficiencies (*I*_n_ > 0.1) in Additional file 1: Table S3.

### Population genetic structures revealed by ***F***_ST_ estimations and ADMIXTURE analysis

**Fig. 3:**
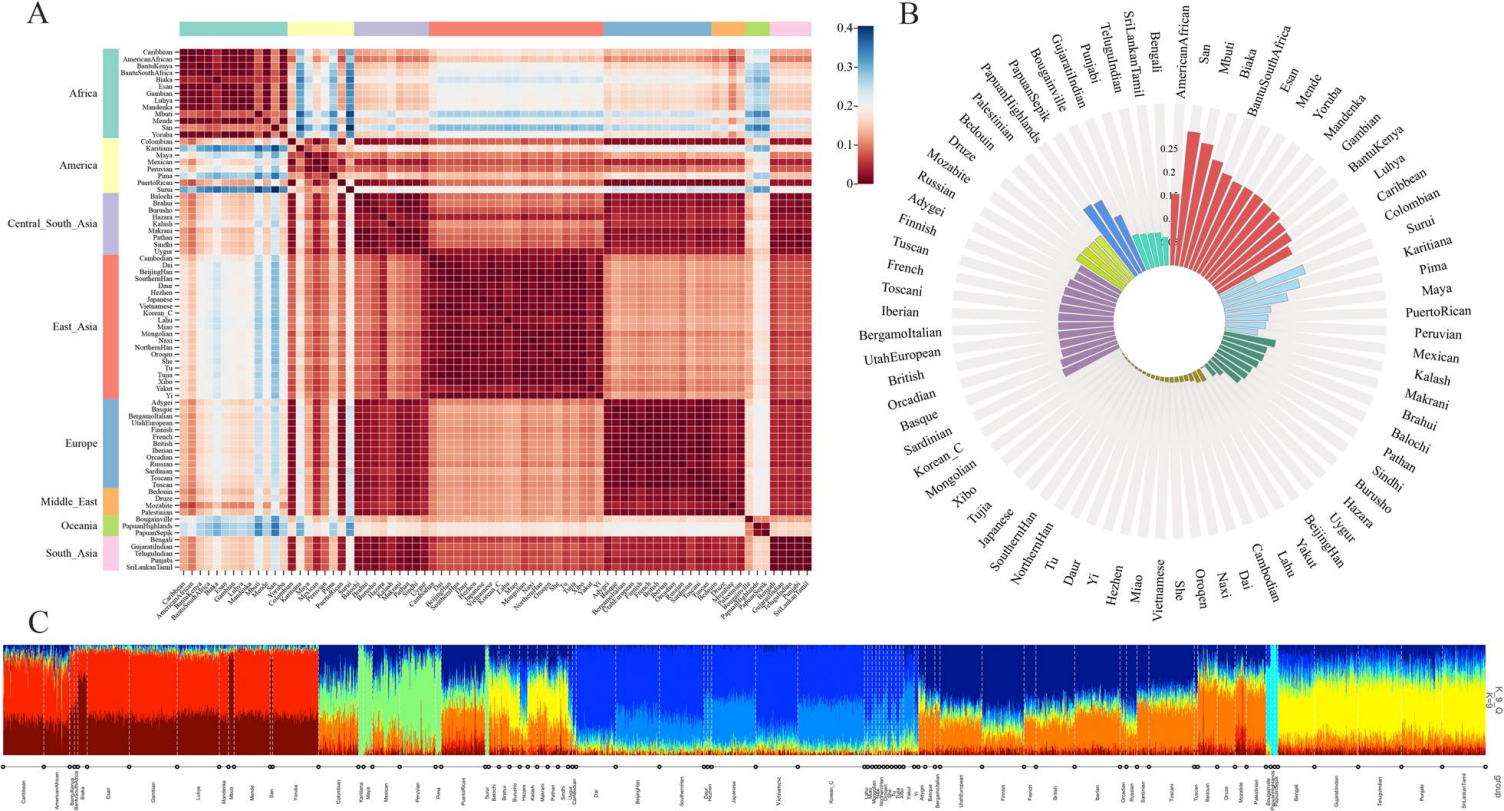
Pairwise *F*_ST_ values among different populations. **A** Heat map of the *F*_ST_ values estimated among different intercontinental populations, including Africa, Europe, East Asia, South Asia, America, Central South Asia, Middle East and Oceania populations. The color ranges from red to blue, corresponding to *F*_ST_ values from low to high. **B** Bar plot of the *F*_ST_ values between the Chinese Korean ethnic group and the reference populations. Populations labeled with the same color indicate they are from the same continents; **C** ADMIXTURE results for *K* = 9. The genetic components of different populations are represented in different colors

The fixation index (*F*_ST_) was used to estimate pairwise genetic differentiations among the overall 76 populations, encompassing the Chinese Korean ethnic group genotyped in this study and 75 worldwide populations selected from the 1 KG and the HGDP. First, we computed the pairwise *F*_*ST*_ among different intercontinental populations and visualized the *F*_ST_ values using a heatmap. Then, we extracted the *F*_*ST*_ between the Chinese Korean ethnic group and its reference populations from different continents to visually present genetic relationships among these populations. Our observations unveiled significantly lower genetic distinctions among populations residing within the same continents, whereas higher genetic differences were evident when comparing populations from different continents. The African populations were relatively more genetically far from other intercontinental populations (Fig. 3A). In this context, the Chinese Korean ethnic group exhibited the most genetic differentiations from African populations, as indicated by a mean *F*_ST_ of 0.2138. In contrast, the Chinese Korean ethnic group shared the least genetic divergence from the East Asian populations, with a mean *F*_ST_ of 0.0087. Meanwhile, the mean *F*_ST_ values between the Chinese Korean ethnic group and populations from South Asia, Central South Asia, America, Europe, Middle East and Oceania populations ranged from 0.0714 to 0.1637, lower than those observed for African populations but higher than those for East Asian populations (Fig. 3B). In the East Asian reference populations, the Yakut and Lahu groups showed the most genetic differentiations from the Chinese Korean ethnic group compared with other East Asian populations. In contrast, the Han populations from various regions (Northern Han, Southern Han, Beijing Han), Xibo, Tujia, Japanese, and Mongolian groups displayed the least genetic differentiations from the Chinese Korean ethnic group. Generally, the *F*_ST_ values of pairwise populations increased relative to the magnitude of geographical separation. Detailed information on the F_*ST*_ values of the pairwise populations was shown in Additional file 1: Table S4.

The ADMIXTURE analysis was performed to estimate the genetic structure of the Chinese Korean ethnic group and compared it to the 75 reference populations. This investigation allowed us to better understand the ancestries of different populations by separating their genotype data into distinct components, assuming between two and ten ancestral populations in running the ADMIXTURE program. When *K* = 9, the least CV error was determined. As shown in Fig. 3C, the primary ancestral element found in Korean ethnic genomes corresponded to the genetic heritage of East Asian populations, as the *K* increased from two to ten, which supported the finding that the Chinese Korean ethnic group was closely related to East Asia populations. The results of ADMIXTURE analyses for *K* values from two to ten was shown in Additional file 1: Fig. S3.

### Principal component analysis

As anticipated from the *F*_*ST*_ results, the Chinese Korean ethnic group showed the least genetic differentiations from East Asia populations compared with non-East Asia populations. However, we were still unaware of any potential population substructures within the East Asia populations. To reveal the genetic relationships between the Chinese Korean ethnic group and the East Asia reference populations, we performed the PCA. We tested the power of this panel for disclosing population structures in a stepwise method, by generating the population-level and individual-level PCA plots, respectively. The population-level PCA (Additional file 1: Fig. S4A) showed that distinct population clusters were generated concordant with their geographic locations, especially for Africa, Europe, South Asia and East Asia populations. More dispersive distributions were observed for America, Central South Asia, Oceania, and Middle East populations. Focusing the PCA on East Asia populations (as shown in Additional file 1: Fig. S4B), it became easier to discern the genetic affiliations among the East Asian populations. Nevertheless, the genetic structure of the Chinese Korean ethnic group exhibited a stronger resemblance to the Han Chinese hailing from diverse regional origins, as well as Tujia, Yi, and Japanese populations.

**Fig. 4:**
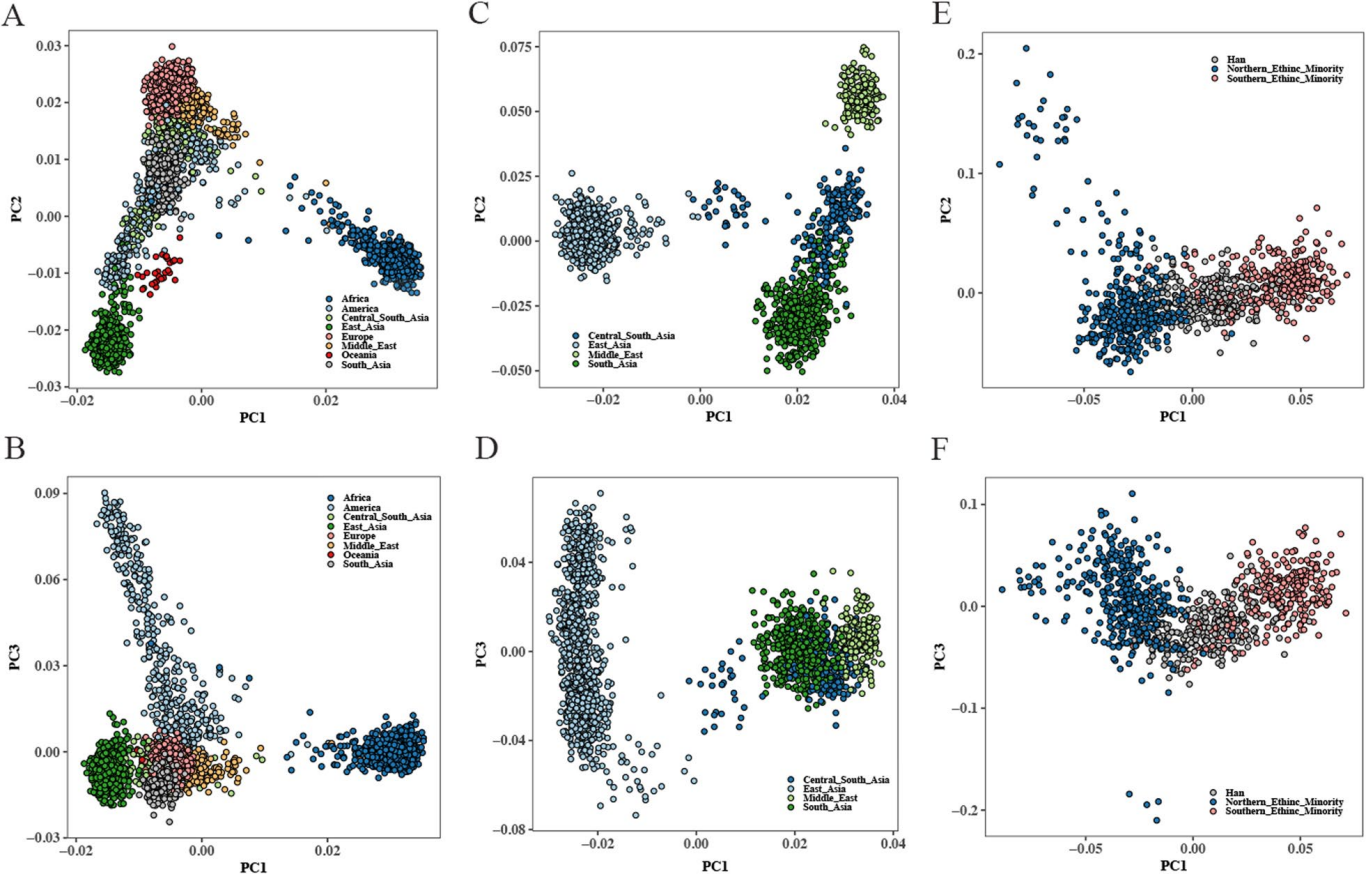
Population genetic structures revealed by principal component analyses. Each dot represents a single individual and is colored according to its continental origin. **A**, **B** PCA of the overall individuals with the first three principal components (PC) involved, which reveals the population genetic structures of the intercontinental populations from eight major global regions; **C**, **D** PCA of the individuals from East Asia, South Asia, Central South Asia and Middle East with the first three PCs involved, which reveals the population genetic structures of these populations; **E**, **F** PCA of the individuals from East Asia with the first three PCs involved, which reveals the population genetic structures of East Asia populations. The individuals from East Asia are categorized into the Han populations, northern ethnic minorities and southern ethnic minorities

Next, we verified the ability of this panel to make ancestral inferences for individuals of unknown origin by individual-level PCA. We demonstrated that individuals of different intercontinental origins could be clustered according to their biogeographic locations or genetic structure similarities (Fig. 4A, B), consistent with the population-level PCA results. We also recreated the PCA plots involving South Asia, Central South Asia, Middle East, and East Asia populations to further inspect the efficiency of this panel in distinguishing populations sharing closer geographic proximity. As indicated in Fig. 4C, D, four distinct population clusters were generated: South Asia, Central South Asia, Middle East, and East Asia population clusters. The Middle East and the Central South Asia clusters were relatively distant from the East Asia and South Asia clusters, reaffirming that genetic similarities decrease as geographical separations increase. When the PCA was restricted to East Asia individuals, there was a distinct separation among the Han populations, the Northern ethnic minorities, and the Southern ethnic minorities (Fig. 4E, F), suggesting that genetic substructure may still exist in some geographically closed populations. The results of individual-level PCA further supported the conclusion that this panel could be potentially useful for ancestry inference, capable of distinguishing not only between distant populations but also among Han Chinese and the northern and southern minority groups in East Asia.

### Gene flow among different intercontinental populations

**Fig. 5:**
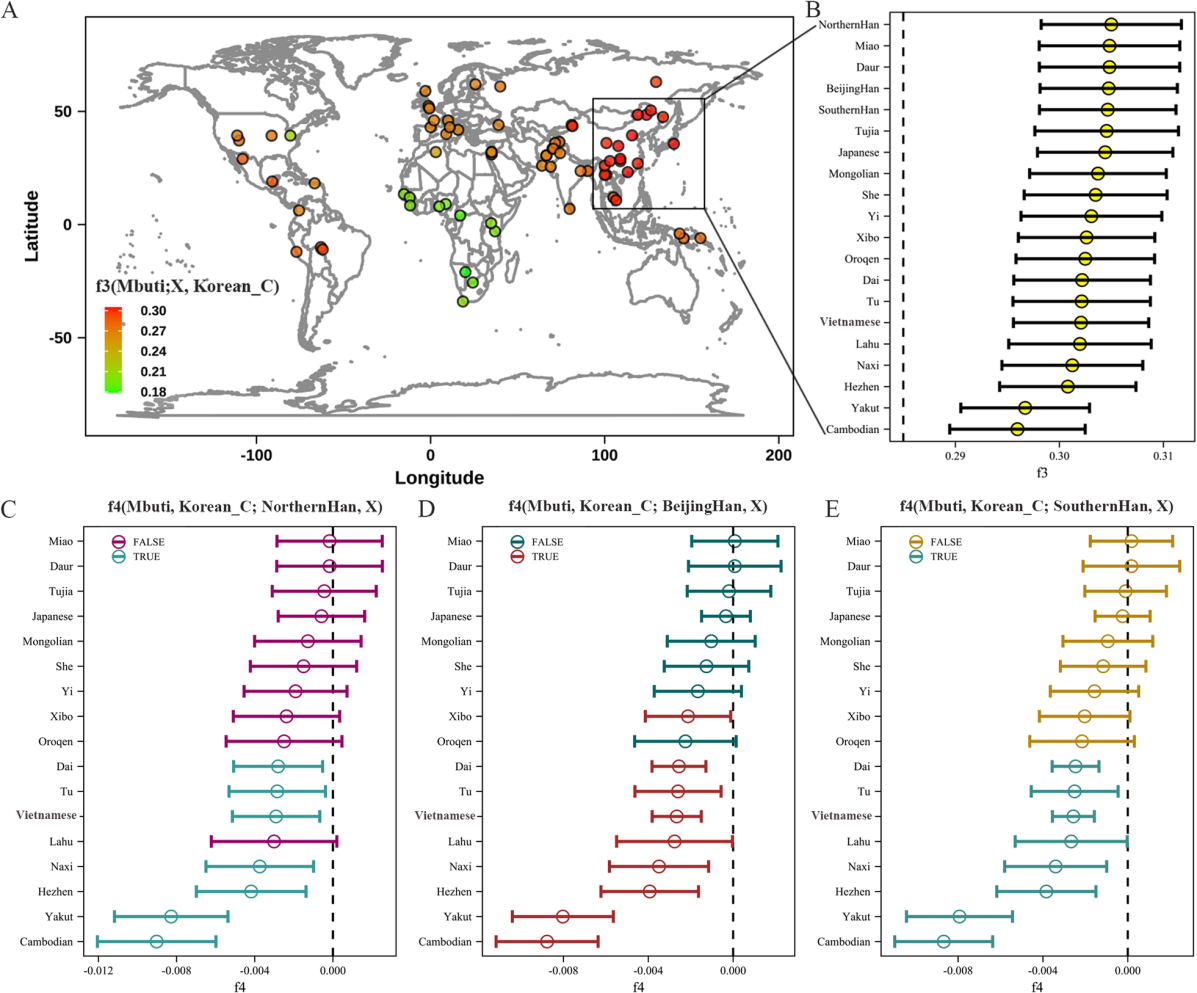
Gene flow estimated among the Chinese Korean ethnic group and the reference populations. **A** Pairwise outgroup-*f*_3_ of the Chinese Korean ethnic group and the reference populations. The color gradient ranges from green to red, corresponding to outgroup-*f*_3_ values from low to high. The map shows the approximate geographic distribution for each population; **B** Pairwise outgroup-*f*_3_ of the Chinese Korean ethnic group and the East Asian reference populations; **C**–**E** Distribution of *f*_4_ values from Dstats tests under the model of [Mbuti, Korean_C; X, Y], where *X* represents the Han populations of different regions, *Y* represents different East Asian populations and the Mbuti population serves as an outgroup

Compared to previous reports, this study allowed us to better examine ADMIXTURE and the recent gene flow within the Chinese Korean ethnic group and the reference populations through computation of the f statistics by enlarging the sample size and the number of SNP loci. As indicated in Fig. 5A, the outgroup-*f*_3_ statistics showed that the 74 populations (except for the Korean_C and the Mbuti populations) could be separated into different genetic clusters based on pairwise shared genetic drift. The Chinese Korean ethnic group shared the most genetic drifts with the East Asia populations, followed by South Asia, Central South Asia, Middle East, Europe, America, Oceania and Africa populations. Furthermore, we extracted the outgroup-*f*_3_ statistics calculated in the East Asian populations to determine the relationships between the Chinese Korean ethnic group and the other East Asian reference populations. As shown in Fig. 5B, the Northern Han, Miao, Daur, Beijing Han, Southern Han, Tujia, Japanese and Mongolian shared more genetic drifts with the Chinese Korean ethnic group, reconfirming pairwise *F*_*ST*_ and PCA conclusions. We also used the *f*_4_ statistics to assess the shared genetic ancestries or admixtures among the East Asia populations. The *f*_4_ statistics showed significantly more genetic exchanges between the Chinese Korean ethnic group and the Northern Han, Beijing Han, and Southern Han populations compared to Dai, Tu, Vietnam, Naxi, Hezhen, Yakut, and Cambodian populations (*f*_4_ < 0, *Z* > 2) (Fig. 5C–E). However, when the Miao, Daur, Tujia, Japanese, Mongolian, She, Yi and Xibo populations were considered as the comparison populations, no significant genetic exchanges were observed between the Chinese Korean ethnic group and the Northern Han, Beijing Han, and Southern Han populations (*f*_4_ < 0, Z < 2), suggesting that the Chinese Korean ethnic group might share more genetic affinities with Han populations across different regions and with the Daur, Tujia, Japanese, Mongolian, Miao, She, Yi and Xibo populations.

### Phylogeny reconstruction of the overall populations using diverse algorithms

**Fig. 6:**
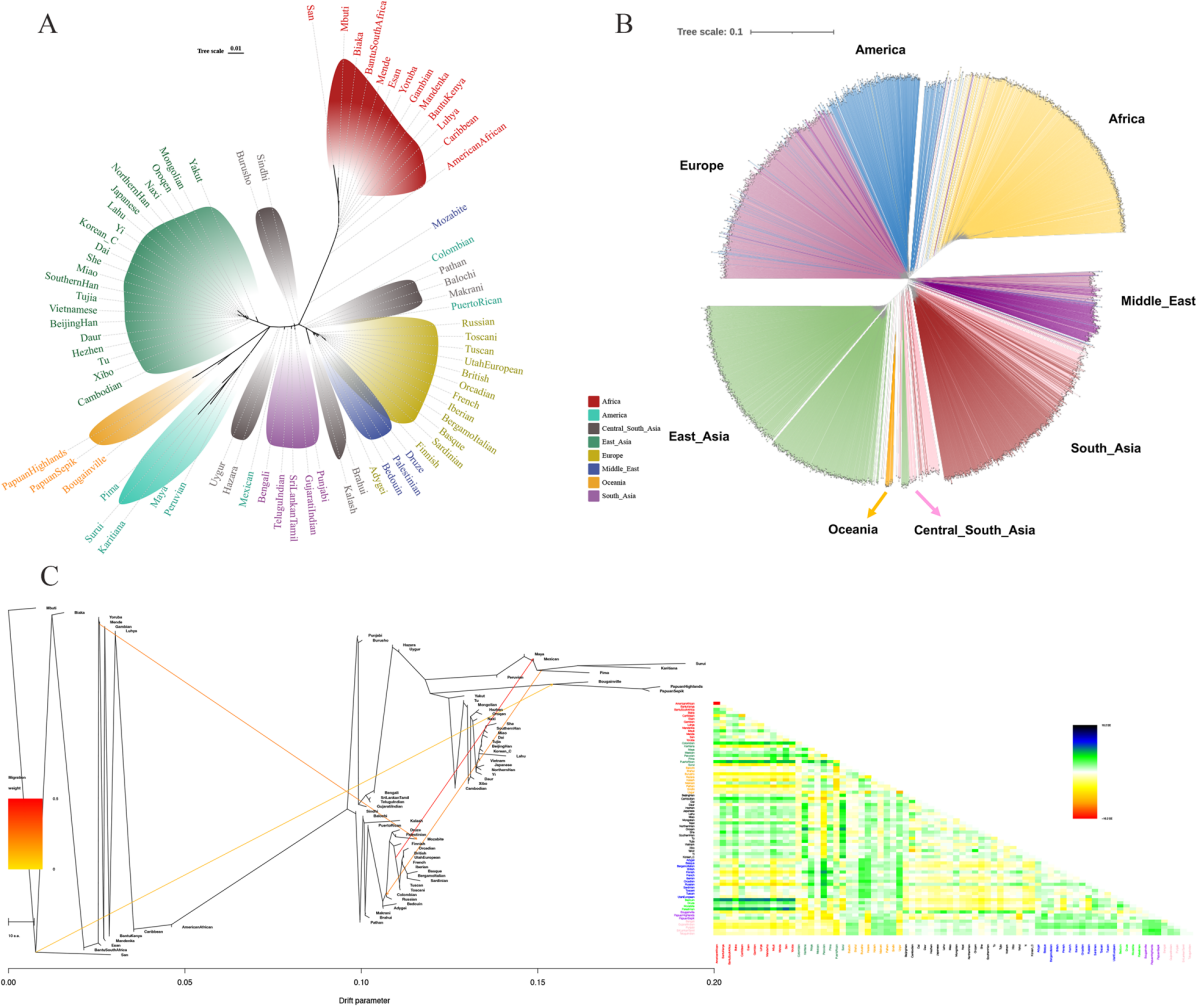
Phylogenetic relatedness of the Chinese Korean ethnic group and the reference populations. **A** Population-level maximum-likelihood phylogenetic tree; **B** Individual-level maximum-likelihood phylogenetic tree; **C** Population-level maximum likelihood tree and pairwise residuals for the phylogenies after accounting for four migration events. The scale bar shows the average standard error of the entries in the covariance matrix

It is widely acknowledged that modern humans originated from an African population, making it a useful starting point for analyzing evolutionary relationships and interpreting the genetic tree. At this point, we inferred the unrooted phylogenies for all 76 populations based on both pairwise *F*_*ST*_ values (Fig. 6A) and the genotype data of high-density SNP loci (Fig. 6B), respectively. The results indicated that populations from the same continents shared a subbranch in the population-level tree (Fig. 6A). The Chinese Korean ethnic group was notably positioned within the East Asia cluster. We could easily distinguish seven major population clusters, representing Africa, Europe, East Asia, South Asia, Middle East, America, and Oceania populations. In contrast, the Central South Asia populations were dispersedly distributed in the phylogenetic tree. However, the individual-level phylogenetic tree showed compelling evidence of closer phylogenetic relationships among some Americans and Europeans, Europeans and Middle East individuals, South Asians and Central South Asians (Fig. 6B).

We also applied a model based on population allele frequencies to account for variances introduced by secondary migration events to test whether admixture was confounding the phylogeny. The maximum likelihood (ML) trees with one to seven admixture events (∆*M* = 1–7) were generated for 20 iterations by the TreeMix software. Based on the distributions of *∆M*, the optimal number of admixture events for the Chinese Korean ethnic group and the reference populations was indicated to be ∆*M* = 4. The ML tree, considering a total of 76 populations along with four admixture events (∆*M* = 4), was shown in Fig. 6C. These results showed that Africa, East Asia, Europe, and South Asia populations consistently clustered together, forming distinct branches in the tree. In contrast, the Central South Asia, Oceania, and Middle East populations did not exhibit a strong tendency to form distinct clusters. In this study, the gene flow events basically occurred from African populations to American, European, and Oceanian populations. When up to seven migration events were included in the model, we further detected admixture from European to American populations. However, we did not detect direct admixture between the Chinese Korean ethnic group and reference populations even after allowing for seven migration events. According to the phylogenetic reconstruction and admixture event estimation analyses, we showed that the Chinese Korean ethnic group was genetically more similar to the East Asia populations. Besides, the gene pool of the Chinese Korean ethnic group was relatively less influenced by contributions from other intercontinental origins. The results of Treemix analyses and pairwise residuals for one to seven migration events were shown in Additional file 1: Fig. S5~6.

## Discussion

The development of NGS technology has revolutionized forensic DNA investigation. The advantages of NGS to forensic DNA analyses can be summarized as follows: (1) increased sensitivity, (2) simultaneous analysis of multiple markers, (3) enhanced discrimination power, (4) expanded data output, (5) adaptability to degraded samples, (6) improved efficiency, and (7) the ability to provide comprehensive genetic profiling. The Illumina sequencing platform has gained significant popularity and acceptance in forensic applications, primarily due to its high throughput, accuracy, and a broad range of sequencing capabilities. In recent years, the MGI has independently developed a sequencing platform that builds libraries based on DNA nanospheres and applies a rolling loop replication strategy for sequencing. Studies have shown that the MGI sequencer has many advantages such as high accuracy, low duplicate sequence rate and low label jump rate, rendering it a powerful tool for forensic DNA practice. Additionally, a previous study has indicated that the 1993-SNP panel, developed on the MGI sequencer, could identify complex relatives in the Han Chinese population [21]. However, the potential applications of the 1993-SNP panel in Chinese ethnic minorities remain largely uncharacterized.

Based on the above considerations, we further verified the efficiency of the 1993-SNP panel for various forensic applications in the Chinese Korean ethnic group from the Yanbian Korean Autonomous Prefecture, China. First, the genetic diversities of the 1993 SNP loci and the potential applications of this new panel for individual identification and parentage testing were investigated by calculating a series of forensic parameters. Then, we integrated the typing data of the Chinese Korean ethnic group with data derived from reference populations representing eight different continents worldwide, to provide an extensive exploration of the genetic background of the Chinese Korean ethnic group. To the best of our knowledge, the number of Chinese Koreans and the SNP loci covered in this study surpasses barely all previous studies on the genetic characterization of the Chinese Korean ethnic group. This study was conducted to further expand the genetic diversity profile of the Chinese Korean ethnic group, which will not only serve as a valuable resource for future genome-wide association studies concerning the Chinese Korean ethnic group but also extend insights into its neighboring populations across East Asia.

We have confirmed that this new panel can be effectively used for individual identification and parentage testing in the Chinese Korean ethnic group. Considering the potential challenges of SNP locus detection in aged or degraded DNA samples, we evaluated the trend of 1-CPD and 1-CPE values as the number of SNP loci increased. The results showed that even with only 10% of the loci detected (200/1993), the threshold of statistical determination required for individual identification and paternity testing can still be reliably met. By simulating different degrees of kinships separately, we confirmed that this panel could effectively determine parent–child, full-sibling, half-sibling and grandparent-grandchild kinships from unrelated individuals, indicating that this panel was potentially useful for complex kinship determinations. However, this conclusion should be further validated with real cases in future studies.

This study expanded the population analyses to a global scale by incorporating 75 reference populations from eight different geographic regions. In the population genetic analyses, we showed that the Chinese Korean ethnic group was genetically more related to the East Asian populations in comparison to groups outside the East Asian context. More specifically, the Chinese Korean ethnic group has been shown to be closely related to the Han Chinese populations and several ethnic minorities residing in northern China. The influence of the geographic location in the differentiation of different intercontinental populations was observed in the PCA, which defined major clusters coincident with Africa, Europe, East Asia, South Asia, Middle East, Central South Asia, and Oceania populations. Pairwise *F*_*ST*_ among different populations also supported that the genetic affinities of populations decreased with increased geographic distances, consistent with the isolation-by-distance model [32]. This geographic pattern was also observed in the ADMIXTURE analyses in which nine genetic components were identified in the Chinese Korean ethnic group and the overall 75 reference populations. With the increase of the pre-assumed ancestral components, the genetic composition of the Chinese Korean ethnic group remained consistent with the East Asian populations. Phylogenetic reconstruction and ADMIXTURE analyses also revealed that the Chinese Korean ethnic group shared more genetic similarities with East Asian populations. Furthermore, there was no detectable influence of genetic ancestries from other intercontinental populations on the gene pool of the Chinese Korean ethnic group.

In recent years, the genomic characteristics of the ethnic Korean population in South Korea has been well characterized by a series of genome-wide studies. The Koreans in South Korea were discovered to share relatively close genetic connections with other East Asian populations, including the Japanese and the Han Chinese [33–35]. In 2022, Lee et al. studied 1896 whole-genome sequences and 3409 whole-exome sequences from healthy individuals of Korean ethnicity in South Asia and found that the ethnic Korean population in South Asia might have undergone a recent divergence and continuous admixture with its neighboring populations in East Asia, like CHB and JPT in the 1 KG [36]. Apart from genome-wide analyses, nucleotide sequences of the major noncoding (D-loop) region of human mitochondrial DNA have revealed that the ethnic Korean population in South Asia might share greater genetic connections with Manchurians, Japanese, Mongolians, and northern Han Chinese [37]. In this study, we showed clear evidence that the Chinese Korean ethnic group could be closely related to Han Chinese populations across different regions, as well as the Miao, Daur, Tujia, Japanese, Mongolian and Xibo populations. Compared with previous studies, we not only reconfirmed the closer genetic relationships between the Koreans, Han Chinese and Japanese, but also we revealed that some ethnic minorities of northern origin may also share more genetic similarities with the Korean ethnic group in China.

To summarize, the genomic data and findings yielded in this study enhance our understanding of East Asia population histories and contribute to the broader knowledge of global genetic history. Importantly, our data have provided a crucial resource and biomedical reference that will facilitate understanding of rare and common genetic variants in the Chinese Korean ethnic group. Meanwhile, we also admitted that given the limited number of participants included in this study, there may be some uncertainty in the conclusions drawn when exploring the genomic background of the Korean ethnic group in China. In future research endeavors, we intend to enhance our analysis of the genetic structure of the Chinese Korean ethnic group by including a more extensive cohort of Korean individuals, as well as its neighboring ethnic groups that may engage in significant genetic exchanges [38].

### Supplementary Information

**Additional file 1. Fig. S1:** Sequencing depths and allele coverage ratios of the SNP loci in the Chinese Korean ethnic group. **A** Distribution of the 1993 SNP loci in the 22 autosomes; **B** Histogram of the average sequencing depths for 1993 SNP loci in 161 Chinese Korean individuals; **C** Boxplot of the sequencing depths for SNP loci with a sequencing depth less than 500×; **D** Histogram of the average allele coverage ratios for the heterozygous SNP loci in the Korean ethnic group. **Fig. S2:** Forensic efficiencies of the 1946 SNPs in the Chinese Korean ethnic group. **A** Forensic statistical parameters, encompassing gene diversity (GD), H_obs_ (Observed heterozygosity), PD (Power of discrimination), PE (Power of exclusion) and PM (Probability of match) of the 1946 SNPs; **B** Distribution of 1-CPD and 1-CPE values estimated with the increase of SNP loci. **Fig. S3:** ADMIXTURE results for K = 2 ~10, with the K denoting the pre-assumed ancestry components represented by different colors. The ancestry composition of each population is proportional to the height of different colors. **Fig. S4:** Principal component analyses (PCA) of the Chinese Korean ethnic group and the reference populations. Each dot represents a single individual and is colored according to its continental origin. **A** PCA of the Chinese Korean ethnic group and all the reference populations from eight major geographic regions worldwide. The Chinese Korean ethnic group and East Asian populations are marked with black box; **B** PCA of the Chinese Korean ethnic group and the East Asian reference populations. **Fig. S5:** Phylogenetic reconstruction of Treemix results for one to seven (except for four) migration events between the Chinese Korean ethnic group and the reference populations. **Fig. S6:** Pairwise residuals of Treemix results for one to seven (except for four) migration events between the Chinese Korean ethnic group and the reference populations.

## Data Availability

All data needed to evaluate the conclusions in the paper are presented in the paper and associated supplementary materials. Additional data and materials used in this study may be requested from the authors.
