# Supplementary material for: Targeted sequencing of high-density SNPs provides an enhanced tool for forensic applications and genetic landscape exploration in Chinese Korean ethnic group
Source: Hum Genomics. 2023 Nov 27;17:107. doi: 10.1186/s40246-023-00541-0 (PMC10680316; doi:10.1186/s40246-023-00541-0)
Supplement: Supplementary file 1 — Additional file 1. Fig. S1: Sequencing depths and allele coverage ratios of the SNP loci in the Chinese Korean ethnic group. A Distribution of the 1993 SNP loci in the 22 autosomes; B Histogram of the average sequencing depths for 1993 SNP loci in 161 Chinese Korean individuals; C Boxplot of the sequencing depths for SNP loci with a sequencing depth less than 500×; D Histogram of the average allele coverage ratios for the heterozygous SNP loci in the Korean ethnic group. Fig. S2: Forensic efficiencies of the 1946 SNPs in the Chinese Korean ethnic group. A Forensic statistical parameters, encompassing gene diversity (GD), Hobs (Observed heterozygosity), PD (Power of discrimination), PE (Power of exclusion) and PM (Probability of match) of the 1946 SNPs; B Distribution of 1-CPD and 1-CPE values estimated with the increase of SNP loci. Fig. S3: ADMIXTURE results for K = 2 ~10, with the K denoting the pre-assumed ancestry components represented by different colors. The ancestry composition of each population is proportional to the height of different colors. Fig. S4: Principal component analyses (PCA) of the Chinese Korean ethnic group and the reference populations. Each dot represents a single individual and is colored according to its continental origin. A PCA of the Chinese Korean ethnic group and all the reference populations from eight major geographic regions worldwide. The Chinese Korean ethnic group and East Asian populations are marked with black box; B PCA of the Chinese Korean ethnic group and the East Asian reference populations. Fig. S5: Phylogenetic reconstruction of Treemix results for one to seven (except for four) migration events between the Chinese Korean ethnic group and the reference populations. Fig. S6: Pairwise residuals of Treemix results for one to seven (except for four) migration events between the Chinese Korean ethnic group and the reference populations. [file 40246_2023_541_MOESM1_ESM.zip › Supplementary Materials_upload_new/Supplementary Figure legends.docx]

**Supplementary Figure and Figure legends**


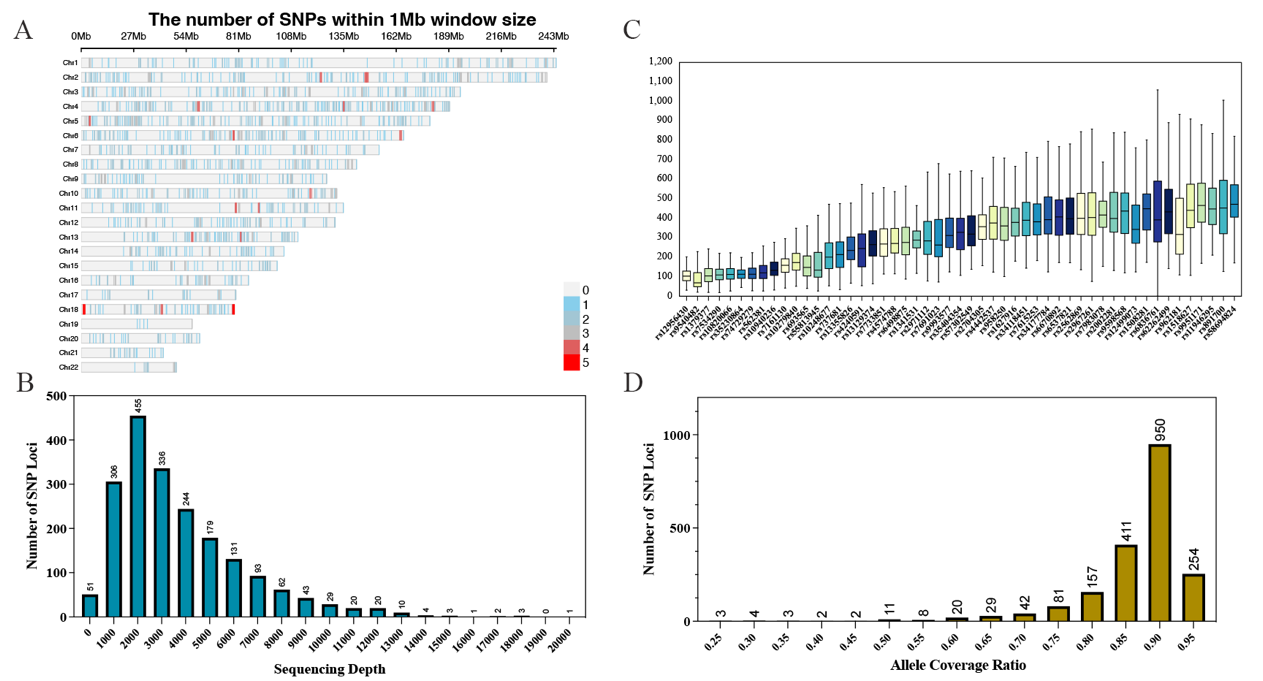


**Fig. S1:** Sequencing depths and allele coverage ratios of the SNP loci in the Chinese Korean ethnic group. **A** Distribution of the 1993 SNP loci in the 22 autosomes; **B** Histogram of the average sequencing depths for 1993 SNP loci in 161 Chinese Korean individuals; **C** Boxplot of the sequencing depths for SNP loci with a sequencing depth less than 500×; **D** Histogram of the average allele coverage ratios for the heterozygous SNP loci in the Korean ethnic group.


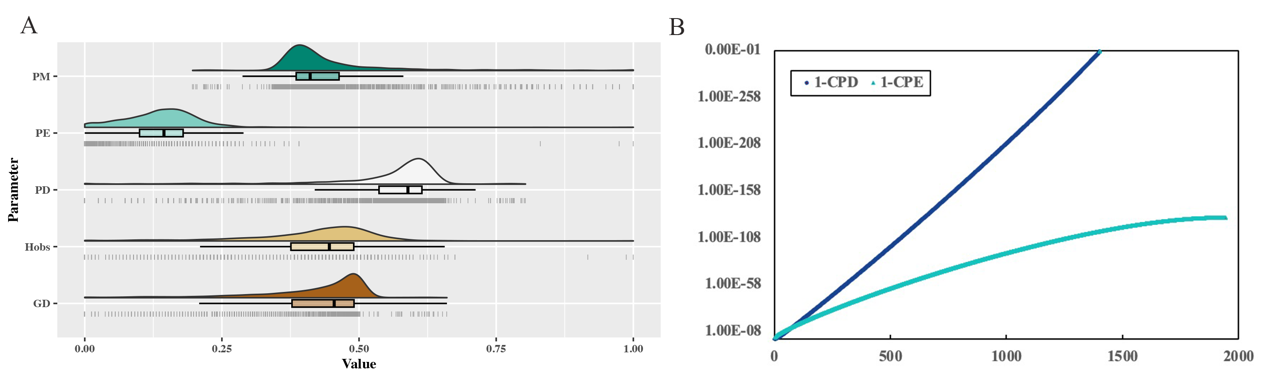


**Fig. S2:** Forensic efficiencies of the 1946 SNPs in the Chinese Korean ethnic group. **A** Forensic statistical parameters, encompassing gene diversity (GD), H_obs_ (Observed heterozygosity), PD (Power of discrimination), PE (Power of exclusion) and PM (Probability of match) of the 1946 SNPs; **B** Distribution of 1-CPD and 1-CPE values estimated with the increase of SNP loci.


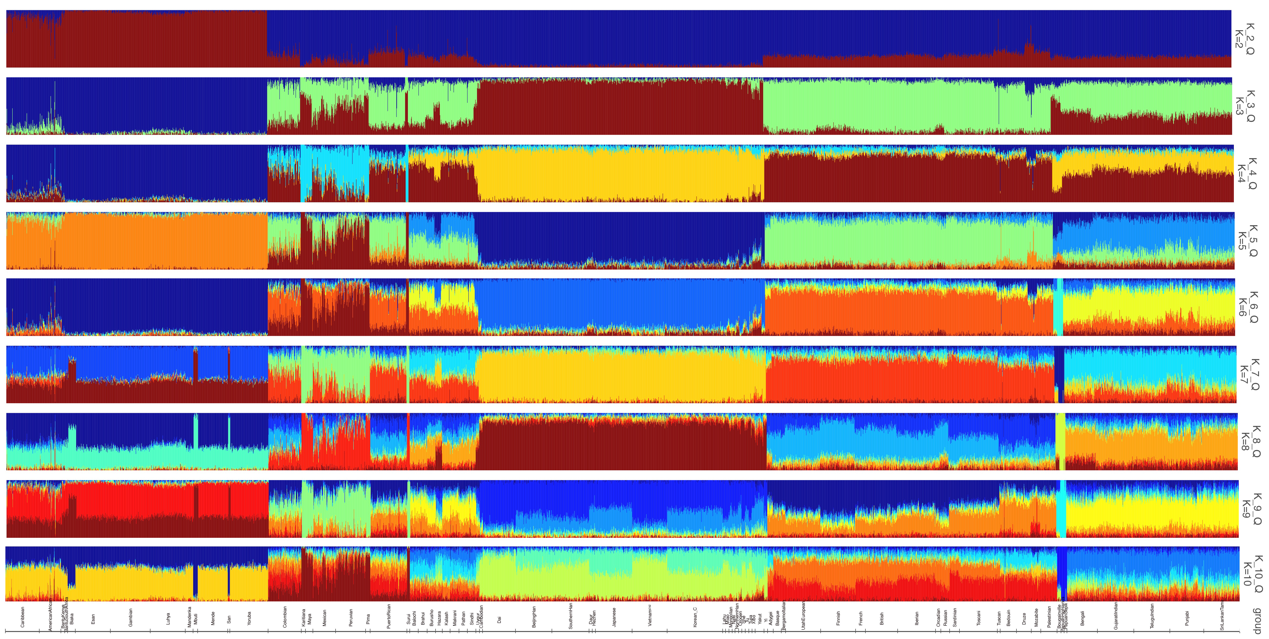


**Fig. S3:** ADMIXTURE results for *K* = 2 ~10, with the *K* denoting the pre-assumed ancestry components represented by different colors. The ancestry composition of each population is proportional to the height of different colors.


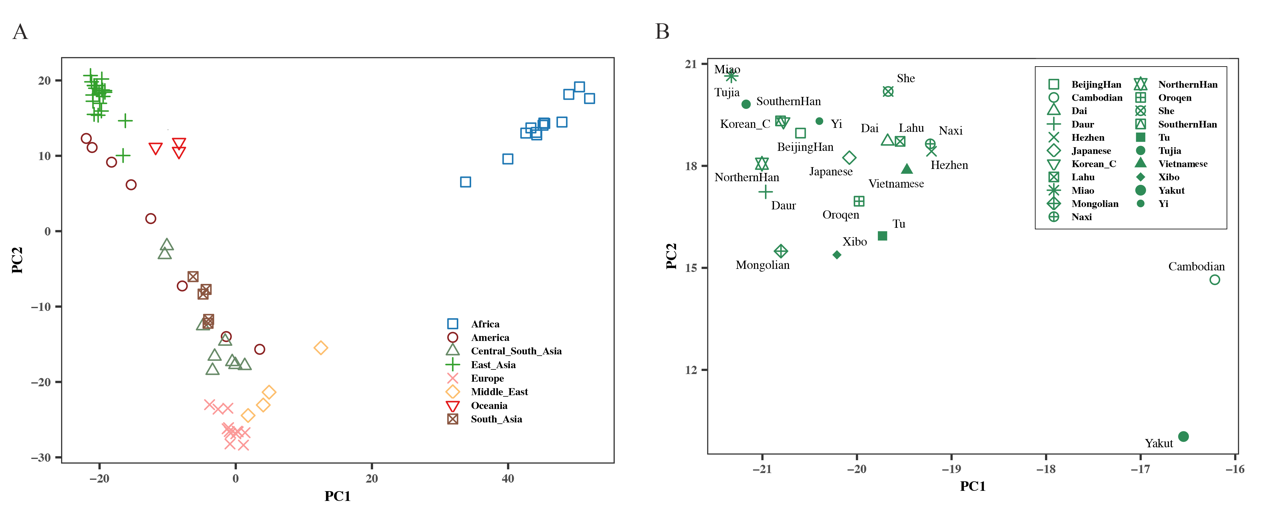


**Fig. S4:** Principal component analyses (PCA) of the Chinese Korean ethnic group and the reference populations. Each dot represents a single individual and is colored according to its continental origins. **A** PCA of the Chinese Korean ethnic group and all the reference populations from eight major geographic regions worldwide. The Chinese Korean ethnic group and East Asian populations are marked with black box; **B** PCA of the Chinese Korean ethnic group and the East Asian reference populations.


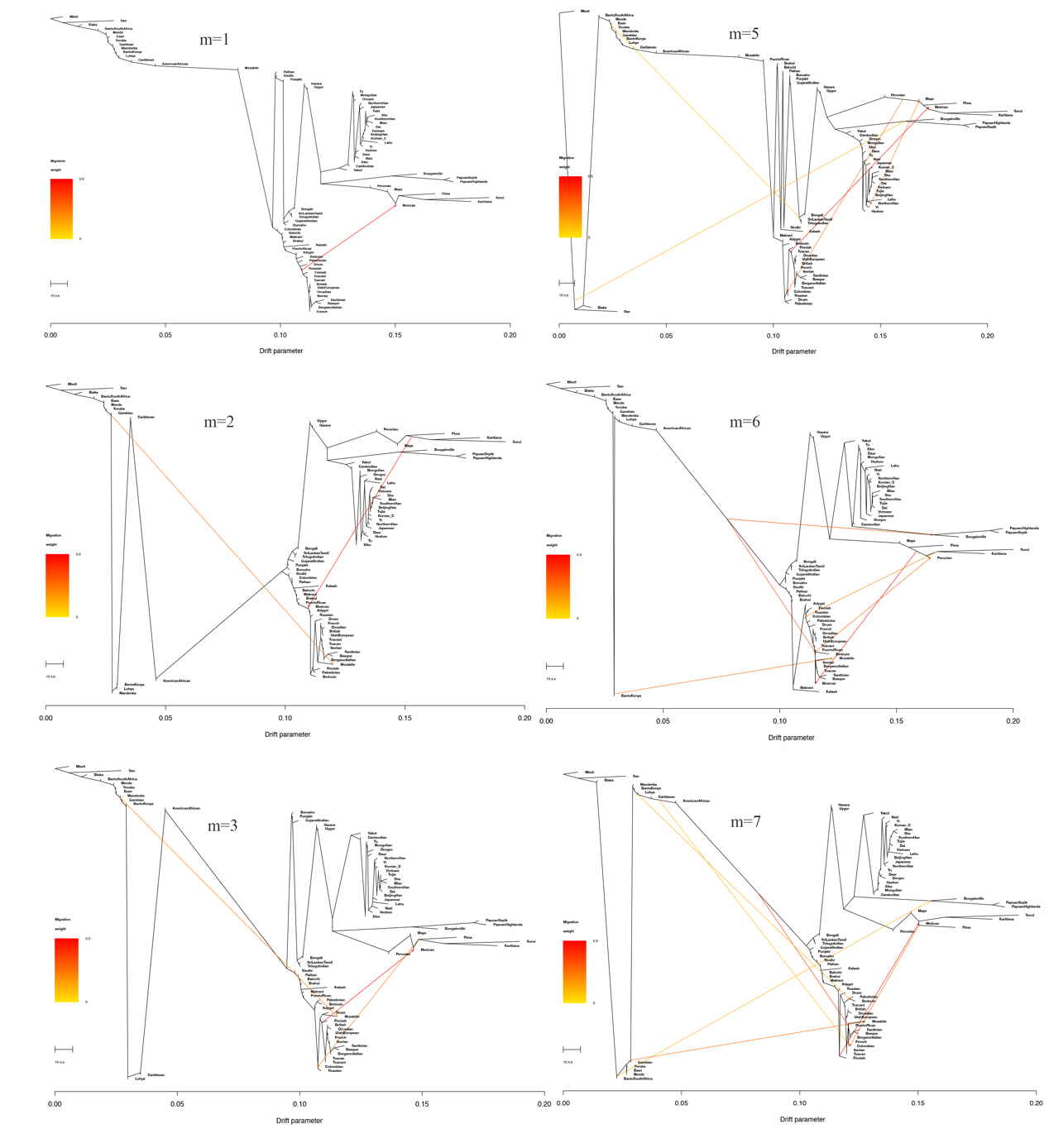


**Fig. S5:** Phylogenetic reconstruction of Treemix results for one to seven (except for four) migration events between the Chinese Korean ethnic group and the reference populations.


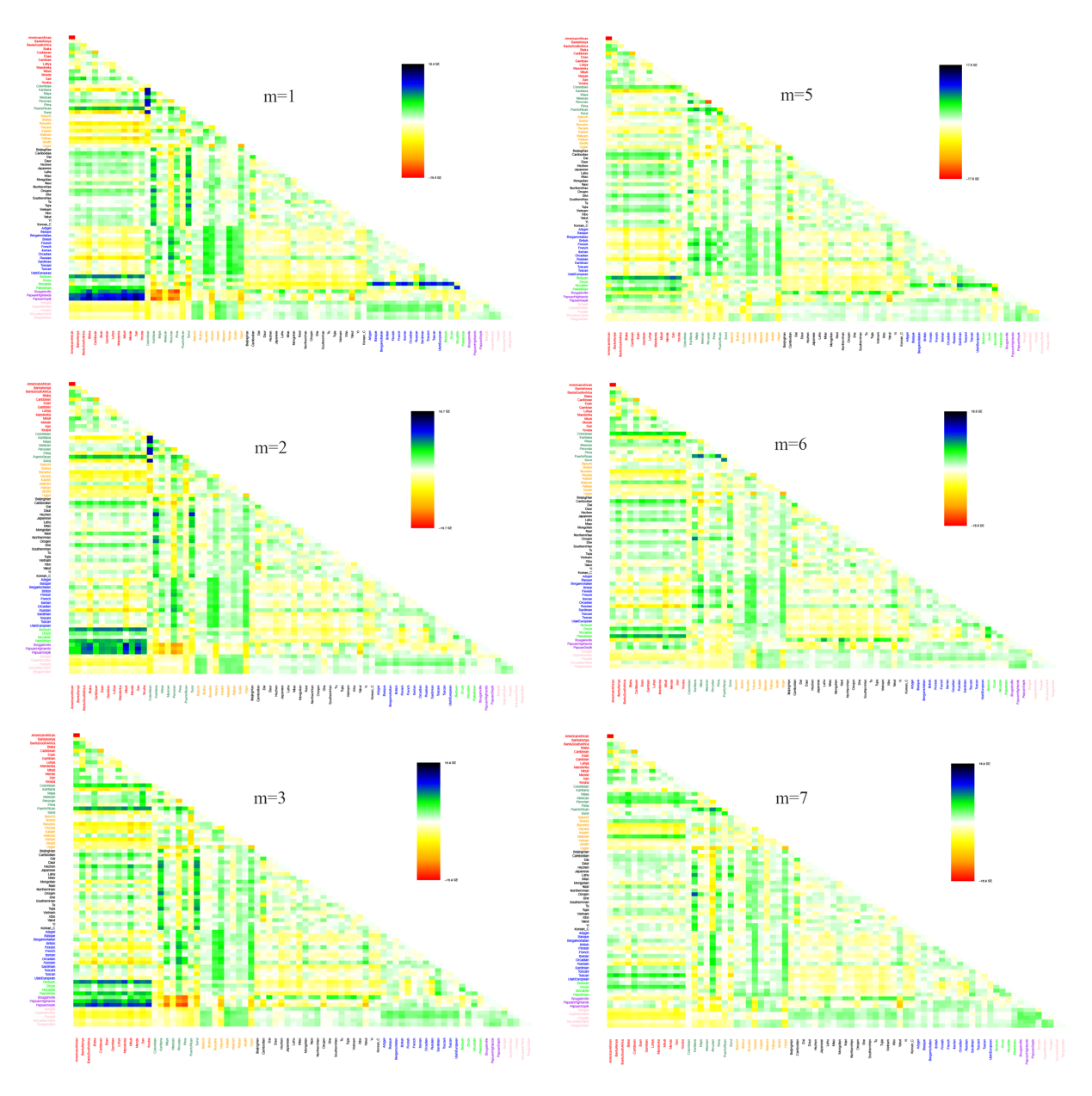


**Fig. S6:** Pairwise residuals of Treemix results for one to seven (except for four) migration events between the Chinese Korean ethnic group and the reference populations.
